# The Efficacy of Strontium and Potassium Toothpastes in Treating Dentine Hypersensitivity: A Systematic Review

**DOI:** 10.1155/2013/573258

**Published:** 2013-04-08

**Authors:** B. F. A. Karim, D. G. Gillam

**Affiliations:** ^1^Dental Institute, Kings College London, Floor 18, Tower Wing, Guys Hospital, London SE1 9RT, UK; ^2^Centre for Adult Oral Health, Institute of Dentistry, Barts and the London School of Medicine and Dentistry, Queen Mary University, London E1 2AD, UK

## Abstract

*Objectives*. The aim of the present paper was to review the published literature in order to identify all relevant studies for inclusion and to determine whether there was any evidence of the efficacy of strontium and potassium toothpastes in the treatment of dentine hypersensitivity (DH). *Methods*. Following a review of 94 relevant papers both from searching electronic databases (PUBMED) and hand searching of relevant written journals, 13 studies were identified, and 7 papers (1 for strontium-based toothpastes and 6 for potassium-based toothpastes) were finally accepted for inclusion. The main outcome measures were the methodology and assessment used by Investigators in studies designed to evaluate DH. *Results*. The results of the present paper would indicate that the reported efficacy of both strontium- and potassium-based toothpastes in relieving DH is questionable. *Conclusions*. The results from the present paper would appear to support the conclusions of previous investigators that there is only minimal evidence for the efficacy of both strontium- and potassium-based toothpastes in relieving symptoms of DH.

## 1. Introduction

Dentine hypersensitivity (DH) is a relatively common dental condition which may have a profound effect on the quality of life of those who suffer with the problem. The main presenting symptom is pain generally in response to cold stimuli. It is also evident from the published literature that DH may be underreported by dental professionals who may have problems in the diagnosis and management of the condition [1, 2]. There are a number of products that have been formulated for either in-office or over-the-counter (OTC) applications, and the mechanism of action of these products appears to work (as evaluated in laboratory-based studies) either on the basis of their tubular occluding properties, for example, restoratives materials such as resins, varnishes, and toothpastes, or by nerve desensitization, for example, potassium-based (chloride, citrate, and nitrate) products [3]. Generally speaking the application of an in-office product may be limited to patients with severe DH limited to one or two affected teeth whereas the recommendation of an OTC product such as a toothpaste or mouthwash may be suitable for patients or consumers with generalized mild to moderate DH [3]. One of the problems when evaluating the efficacy or perceived success of these products is that pain is very subjective and the pain experience may vary from individual to individual [4]. The evaluation of these products is generally conducted by dental professionals in a clinical study that would determine the efficacy or effectiveness of a desensitizing product compared to a placebo, negative, or positive control [5]. The duration of these studies would be determined to some extent as to whether the product was an in-office or OTC product and the clinical claims to be made, for example, instant relief from DH and/or long-lasting relief [5, 6]. There are a number of problems associated with these studies, for example, the variation of the methodology employed and whether they are typical of the stimuli or sensation experienced by patients and consumers in day-to-day experience [4]. A further concern from these studies may be related to whether the study population is truly representative of the individual suffering from DH in the general community. Although there is evidence from the published literature that these products have demonstrated measurable positive improvements in terms of percentage reductions from baseline values, it is difficult to determine the clinical relevance of such reductions in individuals with DH [5, 7–9]. Ideally a true end-point or clinical outcome from these studies would be the complete absence of discomfort following the application of a product in the in-office situation or relief of discomfort over time when using an OTC product that may enable an individual to have an accepted quality of life without the previously perceived discomfort [4, 5]. Currently no universally accepted OTC product that completely relieves the symptoms of DH appears to be available although there are a number of products that have been formulated for the treatment and management of DH which have demonstrated varying degrees of effectiveness. For example, products that have been shown to act as tubular occludents, such as strontium-based toothpastes, Pro-Argin-based toothpastes containing arginine and calcium carbonate, hydroxyapatite and NovaMin (calcium sodium phosphosilicate) toothpastes or products that act as a nerve desensitiser (e.g., potassium-based products) [3, 10–12]. The aim of the present paper was therefore to identify all relevant studies from the available published literature in order to determine whether there was any evidence of the efficacy of a tubular occludent (strontium) and a nerve desensitiser (potassium) toothpaste for the treatment of DH. The objective was also to update the results from previous reviews for potassium-based toothpastes [7–10] and strontium-based toothpastes [11], respectively, in the published literature up to 31st December 2010 using an agreed search protocol based on a modified version of Poulsen et al. [9] and Hsui [13].

## 2. Aim and Objectives

The aim of this paper was to examine the available published literature in order to determine the efficacy of both strontium (Sr) (chloride and acetate) and potassium (K^+^) (nitrate, citrate, and chloride) toothpastes in the treatment of DH. 

## 3. Methodology

The search methodology used for the present study was based on a modified version of Poulsen et al. [9] and Hsui [13] as indicated later.

### 3.1. Selection Criteria

#### 3.1.1. Types of Study

This review included any type of studies (e.g., randomized controlled clinical trials) in which strontium and/or potassium-containing toothpastes/gels were compared to nonpotassium and/or nonstrontium toothpastes.

#### 3.1.2. Types of Participants

Included criteria for the relevant studies were dentate, healthy human adults (at least 18 years of age) with a known history of DH from exposed root dentine surfaces.

#### 3.1.3. Types of Interventions

This includes the daily home use of strontium and/or potassium-containing toothpastes/gels compared to control toothpastes/gels. In each study the toothpastes compared will either both contain fluoride or have no fluoride. The control toothpaste was exactly the same as the test toothpaste apart from the addition of either a strontium or potassium salt.

#### 3.1.4. Types of Outcome Measures

This includes changes in (1) pain symptoms in response to the test procedures, including tactile, thermal, and air blast stimuli, or (2) patients' subjective assessment of pain during their daily experience. Only studies that reported data after 6 and 8 weeks were included in the review.

## 4. Search Strategy

The search strategy included using hand searching or electronic databases (e.g., PUBMED) up to 31st December 2010. The hand searching process also included examining relevant published or incomplete journals in English. The searching key words in PUBMED were (cervical OR tooth OR teeth OR dentin* OR dental) AND (sensitiv* OR hypersensitiv* OR pain*) AND (Efficacy*) AND (random* OR trial OR (randomized controlled trial [pt]) OR (controlled clinical trial [pt]) OR cohort* OR longitudinal* OR “follow up” OR prospective* OR case-control).

## 5. Statistical Analysis

Statistical analysis of data from these studies was not attempted due to the variations in the study design, methodology, study duration, and reporting of the pain response (percentages, VAS scores, or pain categories, etc.).

## 6. Method of the Review (Data Collection and Analysis)

From the titles retrieved in the electronic search all relevant clinical studies and reviews were identified by one of the authors (Belkais Karim [BK]) who then obtained copies of all the relevant studies where available for further consideration. Two reviewers (BK and David Gillam [DG]) determined the quality of the eligible papers and data extraction based on the randomisation procedure, allocation concealment, blinding, and description of any dropouts (withdrawals) [13, 14]. Any differences as to inclusion or exclusion of articles were resolved following discussion between BK and DG. Data extracted from the included and excluded studies was completed on the relevant data extraction forms [13].

Sensitivity from DH was assessed using the following types of measurements: tactile (pressure with a standardised probe) or thermal (heat/cold) stimulation or evaporative (air blast) stimulation. Patients' subjective assessment was also included in the analysis. Only sensitivity measurements recorded after 6 and 8 weeks were included due to the variability in the length of the published studies.

## 7. Results

### 7.1. Overall Description of the Included and Excluded Studies

**Figure 1:**
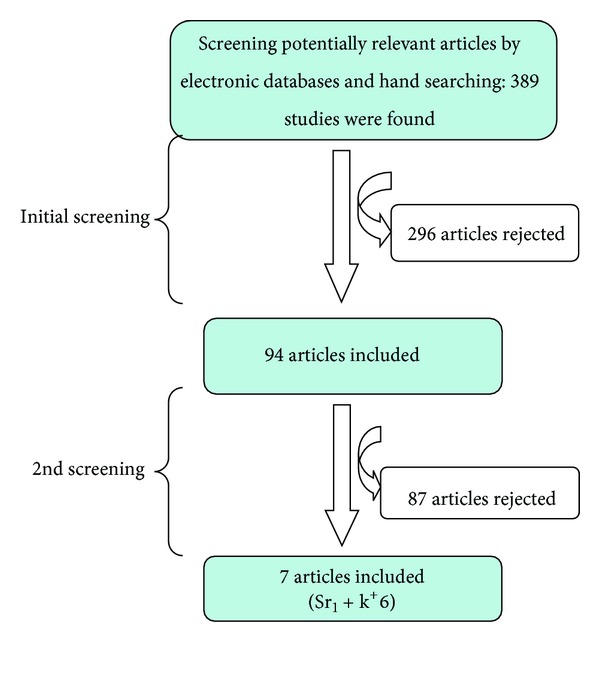
Flow diagram of the study selection process.

After the initial screening of identified articles for the present paper, there were 390 potentially relevant studies found either by searching the electronic databases (PUBMED) or by hand searching articles from the literature. Unpublished articles were found both by searching the electronic databases and by hand searching. 94 studies were regarded as relevant for this study while 296 studies were excluded (Figure 1). Following an evaluation of the selected 94 studies, 87 studies were excluded, 32 of these studies were strontium-based toothpaste studies [7, 15–45] (Table 1), and 55 were potassium-based toothpaste studies [3, 9, 46–98] (Table 2). The reasons for exclusion of these 87 studies are detailed in Tables 1 and 2. 7 strontium- and potassium-based toothpaste studies were included in the present paper [99–105] (Tables 3 and 4). The flow diagram (Figure 1) of the selection procedure is illustrated later.

7 strontium- and potassium-based toothpaste studies therefore fulfilled the criteria for inclusion in the review. In all these studies the experimental toothpaste either contained strontium or potassium whereas the control toothpastes were without strontium or potassium. From the 7 included studies, only 1 article was identified specifically for strontium salts (as the principal (test) toothpaste) and 6 articles were identified specifically for potassium salts (as the principal (test) toothpaste). The description of the 7 included studies is shown in Tables 3 and 4.

### 7.2. Analysis of Included Studies

#### 7.2.1. Study Design

The 7 studies included in the present paper were only from randomised controlled parallel groups blind clinical Trials (RCT). The control toothpastes were either positive (active) [101–103, 105] or negative (placebo) [99, 100, 104]. The blindness was double-blinded [99–105].

#### 7.2.2. Study Population

Most included studies were conducted in either dental practices or university hospitals. The recruited study participants in the included studies were dentate, healthy human adults with a known history of DH. Regarding the gender distribution, most of the included studies enrolled mainly females [99–105]. The total numbers of participants (447) from the 7 included studies were as follows: (1) for the one strontium-based study there were 57 participants and (2) for the six potassium-based studies there were 390 participants, respectively (Tables 3 and 4).

#### 7.2.3. Age Range of Participants

There was variation in the age range distribution(s) in the included studies; however all participants in the included studies were adults (at least 18 years of age). All 7 included studies reported both the age range and the mean age.

#### 7.2.4. Study Duration

The duration of the 7 included studies evaluating the efficacy of strontium- and potassium-based toothpastes/gels in DH was short term (no longer than 3 months), ranging from 8 to 12 weeks (Table 5). According to the Poulsen et al. [9] systematic review, only studies that reported sensitivity measurements following 6 and 8 weeks of product use were included in their 2008 review.

#### 7.2.5. Statistics Power Calculation

There were a wide variety of statistical tests used in the included studies. The most commonly used test was “ANOVA” [99, 101–104] (Table 6).

#### 7.2.6. Randomisation and Allocation Concealment (See [13])

According to Schulz [107] random allocation to intervention groups in a clinical study remains the only method of ensuring that the groups being compared are on an equivalent footing at study outset, thus eliminating selection and confounding biases. In most of the 7 included studies the degree of concealment was unclear (random allocation stated/indicated but the actual allocation concealment method is not described or an apparently adequate concealment scheme is reported but there is uncertainty whether allocation is adequately concealed) [100–105]. In the Minkoff and Axelrod [99] strontium study the randomization process was made externally by a statistical department using a computer-generated random table.

#### 7.2.7. Consideration of Withdrawals and Dropouts (See [13])

According to Bowers [108, 109] withdrawals and dropouts that occur following the randomization process may adversely affect the balance of the two groups that had been achieved through the randomization process which may in turn affect any subsequent data analysis (through loss of data). One way of resolving this problem is to include data of these participants as they were still in the study; this is called *intention-to-treat analysis.* Withdrawals and dropouts were reported in 4 out of 7 included studies (Table 7). 

## 8. Data Analysis

No further analyses were performed on the mean differences from 6 to 8 weeks for any other measurement outcomes for the purpose of meta-analysis.

### 8.1. Previous History of DH Reported at Baseline

This entails any history of DH in the included studies, reported by investigators, in the form of baseline data, which was confirmed by a response to tactile and/or thermal stimulus.

### 8.2. Types of Treatment Intervention

In all the 7 included studies a daily home use of strontium- [99] and/or potassium- [100–105] based toothpastes/gels versus controls (strontium- or potassium-free toothpastes) was the only type of treatment intervention.

### 8.3. The Clinical Methodology Used to Assess DH

The most commonly reported DH/RS assessment methods by investigators in the 7 included studies were tactile (mainly by using a Yeaple probe), thermal (hot/cold air or water), and evaporative (air blast). Tables 3 and 4 show a summary of the characteristics of the included studies including the assessment methods used for DH. Regarding the subjective assessment of DH, VAS was the main subjective scale used [101, 104]. The Nagata et al. study [100] used Tarbet's four-point air sensitivity scale [78, 106]. Only three included studies used the Schiff's cold air sensitivity scale [102, 103, 105]. However, two studies used questionnaires [99, 100] (Table 8).

### 8.4. Calibration and Examiner Training

There was no reported training or calibration for DH, in either the examination or assessment techniques prior to the commencement of the study, in any of the included studies.

### 8.5. Measurement of Compliance

There was no reported measurement of patient compliance, for example, diaries, weighing of toothpastes, or log books in any of the included studies.

## 9. Discussion

It is evident from the published literature that DH is not only a troublesome condition for dental professionals to effectively diagnose and manage but it may also have a profound effect on the quality of life of those who suffer with the problem [110–112]. Currently there is a plethora of remedies available for both OTC and in-office applications; however it is evident that none of these products appear to provide an effective long-lasting solution to the problem [1, 3]. Most of these products either work on the basis of their tubular occluding properties, for example, strontium-based (chloride and acetate) products, or by nerve desensitization, for example, potassium-based (chloride, citrate, and nitrate) products. Evidence for their efficacy however has been questioned by several investigators in a series of reviews over the last decade [7–11, 113].

The aim of the present paper was to evaluate the efficacy of both strontium and potassium toothpastes based on the published systematic review methodology of Poulsen et al. [9]; however the authors concede that the present paper may have been too restrictive in considering only studies with matched placebo controls. It may therefore be argued that the inclusion of studies with a valid negative control, such as a commercially available fluoride toothpaste, may have given a better indication of whether a toothpaste containing the active ingredient could deliver the desired efficacy (e.g., a reduction in sensitivity). The rationale however for conducting the present paper was to determine whether the active ingredient in toothpaste delivers efficacy in the reduction of DH which is the basis of the claims made for these toothpastes by the manufacturers. Other benefits that have been attributed to these toothpastes such as antiplaque and anticaries benefits have not been considered in the present paper. Generally speaking these benefits are often “based on the results of previous caries studies or plaque studies and there is very little evidence from the published literature on desensitising toothpastes that these ingredients have been shown to demonstrate these benefits” [22, 23, 65].

For the purpose of the present paper, studies (e.g., randomized controlled clinical trials) were included in which strontium and/or potassium-containing toothpastes/gels were compared to nonpotassium and/or nonstrontium toothpastes. The type of intervention examined was the daily home use of strontium and/or potassium-containing toothpastes/gels versus control toothpastes/gels. In each study the toothpastes were either both containing fluoride or having no fluoride and the control toothpaste was exactly the same as the test toothpaste apart from the addition of either a strontium (acetate or chloride) or potassium (citrate, chloride, and citrate) salt. It should be noted that currently most strontium-based toothpastes contain an acetate variant rather than the chloride variant and potassium-based toothpastes contain a nitrate variant depending on the particular commercial market.

Following an initial screening of the available publications there were a total of 87 excluded studies following the final filtration of 94 studies (Tables 1 and 2 and Figure 1). The reasons for excluding 32 strontium studies were either due to the different fluoride or abrasive concentrations and compounds in both test and comparison toothpastes [26, 31, 33, 42, 114]. Two studies were excluded as they were related to DH following periodontal treatment (nonsurgical or surgical studies) [16, 41]. A further two studies were excluded due to the short study duration <6 weeks [17, 40]. A further seven studies were also excluded as they were *in vitro* studies [18–20, 25, 32, 38, 39]. Two studies were also excluded since only Sr Cl_2_ solutions/varnishes were used [21, 29]. The Gillam et al. 1992 studies [22, 23] were also excluded as these investigators reported on (1) the effects of Sr Cl_2_ toothpaste on plaque accumulation and/or gingival recession and (2) the effect of different toothpaste abrasives in the test and control toothpastes. One study by Shapiro et al. [36] was excluded as it was a reported abstract article and a further study by Shapiro et al. [37] was also excluded due to the lack of clarity in the reported outcome measures. Two further studies were also excluded due to (1) the lack of randomisation procedures [28] and (2) the lack of a control group [35]. Several review articles were also excluded [7, 24, 27]. Two further studies by Collins and Perkins [44] and Kumar et al. [45] were subsequently excluded by one of the authors (DGG) after the initial filtration of included studies as it was apparent that there were different compounds in the test and comparison toothpastes and no strontium-free control. In summary, 32 strontium-related studies were excluded (Table 1) and only 1 study was included in the present paper (Table 3). The reasons for the exclusion of the 55 potassium-based toothpaste studies were mainly due to the different fluoride concentrations and/or ingredient(s) in the test and comparison toothpastes [49, 50, 54, 56, 68, 79, 82, 88, 97] (Table 2). For example, three studies were excluded since the main aim of these studies was to evaluate the efficacy of KNO_3_ toothpaste/gel in reducing bleaching sensitivity during or following tooth whitening/bleaching procedures [52, 62, 63]. Five studies were also excluded due to the short study duration of the study (<6 weeks) [54, 57, 68, 81, 90]. A further three studies were excluded as these studies were either in-office [51, 73] or *in vitro* studies [76]. Five studies were also excluded as potassium-containing solutions/mouthwashes were used [46, 55, 58, 92, 95]. The Jalalian et al. [66] study was also excluded since this was a study evaluating the efficacy of an KNO_3_ application in reducing DH with full-crown preparations. The study by Pillon et al. [77] was also excluded since the study was based on the results of a single application of 3% potassium oxalate gel immediately following scaling and root planning procedures (SRP). The Tarbet et al. [91] study was also excluded as this study only evaluated the pulpal effect following brushing with a 5% KNO_3_ toothpaste. A further nine studies were excluded since these studies were reported in an abstract [47, 48, 53, 60, 61, 67, 71, 72, 93]. A review by Hodosh [64] was also excluded as well as a non-RCT study reported by the same investigator [65]. A study by Manochehr-Pour et al. [69] was also excluded due to incomplete data reported in the study. The two animal studies by Peacock and Orchardson [74, 75] were also excluded and a pilot study reported by Reinhart et al. [80] was excluded. The studies by Sharma [83], Sharma et al. [84], and Wara-aswapati et al. [94] were also excluded as no data was available at the 6-to-8-week time intervals. Two studies by Silverman [85] and Silverman et al. [86] were excluded either because (1) the test and control toothpastes were not clearly detailed or (2) due to incomplete data recorded in the study. In the Yates et al. [96] study there was no potassium-free comparison group included in the design of the study. The study by Stead et al. [89] was excluded as this was a review paper. Other reviews, by other investigators [24, 70, 78], were also excluded from this paper. A further study by Pradeep and Sharma [98] was subsequently excluded by one of the authors (DGG) as it was apparent that there were different compounds in the test and comparison toothpastes and no potassium-free control. In this study a calcium sodium phosphosilicate toothpaste was evaluated against a positive control potassium nitrate and a placebo without calcium sodium phosphosilicate. In summary, 55 potassium-based toothpaste studies were excluded and the reasons for exclusion were described in Table 2. 

The results from the present paper were therefore based on the 7 included studies (1 strontium-based and 6 potassium-based toothpastes) and in context would appear to support conclusions from the previous reviews that there were measurable positive reductions in DH from baseline values. Although the present review did not include meta-analysis of the published data from these studies for the reasons outlined earlier in the paper, the results from the Poulsen et al. study [9] and the present paper were based on 6 potassium toothpaste studies. The results indicated that these differences were in favour of the treatment group for both “air” and “tactile” measurements but not for the “subjective” measurements. Generally speaking interpreting air, tactile, and subjective elements in DH studies is fraught with difficulties [4, 115]. For example, most studies demonstrate that the placebo group would provide significant improvements in percentage terms as well as the test group and as such any significance between the groups may be masked [7, 42]. The variation in the methodology employed by different examiners (Table 8) may also have an impact on the efficacy of a toothpaste as well as the highly subjective nature of the pain response between individuals [9]. Some investigators in the present paper used a Yeaple or an explorer probe or a thermal probe versus a cold air blast or assessed the subjective assessment using either a visual analogue scale (VAS) or a Schiff scale [101–105] or questionnaires [99, 100, 106] (Tables 4 and 8). An observation when evaluating the methodology reported in the included studies was that there was no reporting of any training or calibration of the investigators prior to the commencement of the studies. Although a number of these investigators (e.g., Schiff [101–103] were experienced assessors in DH evaluation studies it was impossible to determine whether the investigators were consistent in the assessments during the study. It should be noted however that the variability of these subjective pain outcomes is difficult to control even when using objective measures [4].

One of the problems encountered when conducting the present paper was that there was considerable variation with the manner in the studies were designed and conducted, for example, factors such as the duration, variation in sample size, methodology used to assess the products as well as differences in the test, placebo and control toothpastes and the impact of the placebo and non-placebo improvements in the control toothpastes, makes it difficult to make exact comparisons from the results of these studies (Tables 3–8) [7, 9, 11]. There is no doubt that there is a degree of accommodation and awareness of the pain response by individuals during a study as well as confounding variables such as placebo and nonplacebo effects regression to the mean or mode that may subsequently influence the study outcomes. For example, Curro et al. [116] reporting on a series of DH product evaluation studies indicated that there were a number of false positives associated with tactile assessment when using the Yeaple probe particularly at the lower range of pressures. These investigators also highlighted that the range of the placebo effect observed in DH studies is similar to that observed in both medical and pharmaceutical studies.

One of the problems encountered when analysing data from the published literature on the efficacy of strontium and potassium-based toothpastes was that due to the strict inclusion/exclusion criteria based on Poulsen et al. [9] in the present paper none of the included studies made a direct comparison between the two products (Tables 3 and 4). This was a concern and the authors were therefore unable to comment on a direct comparison between the two products and as such may limit the conclusions that could be made regarding the two products. From the published literature it was evident that earlier studies did provide direct comparisons of these products although as Cummins [11] suggested in her review that prior to 1997 there was considerably more variations in the design and conduct of DH studies as well as the ingredients of the toothpastes *per se.* This again may confound any meaningful conclusions when comparing results from these DH evaluation studies. It should be noted however that published studies (after 1997) would appear to follow a similar design and conduct based on the Holland [5] and/or ADA [6] guidelines when assessing various desensitising products. Several investigators have however questioned the validity and reproducibility of some of these techniques for evaluating DH products [4, 115].

The results from the limited number of included strontium-based studies in the present paper would therefore limit any conclusions that may be drawn from the studies (Table 3), even though there is some evidence of their efficacy in a strontium chloride product [17, 21–23, 29, 41] or a strontium acetate product [26, 31, 33, 42, 43, 59]. A number of investigators have also reviewed the efficacy of strontium-based toothpastes [7, 10, 30, 113]. Jackson [7] however indicated in his review that none of the studies on strontium toothpastes demonstrated a consistent, significant improvement in the participants' symptoms of DH when compared with the negative control toothpaste. There also appears to be no supportive evidence from the published literature for strontium salts enhancing the deposition of the ingredients of the toothpaste or increasing the durability of the deposit on the tooth surface [7].

One of the aims of the present review was to update the previous review of Poulsen et al. [9]; however no subsequently published studies (up to 2010) were considered to be suitable for inclusion and as a result no further information on the efficacy of potassium-based toothpastes was forthcoming. A number of investigators have also raised concerns with regard to the efficacy of potassium-based toothpastes and this has led to the suggestion that potassium-based toothpastes may be no more effective than regular fluoride toothpaste [7, 8, 10]. The lack of data on the efficacy of potassium-containing toothpastes in reducing DH has also been highlighted in a recent systematic review by Pol et al. [78].

No conclusions however can be made from the present paper on the direct comparison of the efficacy of strontium and potassium-based toothpastes in reducing DH. This was due to the limitation of any of the included studies making a direct comparison between the two toothpastes although a number of excluded studies did make such a comparison.

## 10. Conclusions

Although both strontium and potassium-based toothpastes have been demonstrated to provide a reduction in clinical symptoms of DH in previously published clinical studies, the conclusions from the present systematic review would suggest that there is insufficient evidence to state categorically whether strontium or potassium salts *per se* are effective in reducing DH.

## Figures and Tables

**Table 1:** Characteristics of Strontium-based toothpastes excluded studies (reasons).

No.	Study	Reason for exclusion
1	Addy et al. [15]	Different abrasive concentrations and compounds in the test and comparison toothpastes
2	Blitzer [16]	The selected participants had developed a general sensitivity during the course of periodontal treatment
3	Carrasco [17]	Study duration was only 20 days (~3 weeks)
4	Dabsie et al. [18]	*In vitro* study
5	Earl et al. [19]	*In vitro* study
6	Gedalia et al. [20]	*In vitro* study
7	Gedalia et al. [21]	The use of SrCl_2_ solution
8	Gillam et al. [22]	Study reported on the effect of SrCl_2_ toothpaste on plaque accumulation and gingival inflammation
9	Gillam et al. [23]	Study reported on the effect of different toothpaste abrasive DH
10	*Goldie [24]	Review (2011)
11	Gutentag [25]	*In vitro* study
12	Hughes et al. [26]	Different fluoride concentrations and compounds in the test and comparison toothpastes
13	*Jackson [7]	Review
14	*Kanapka [27]	Review
15	*Kishore et al. [28]	Not clear if the study was randomised
16	Kobler et al. [29]	SrCl_2_ solution (varnish) was used
17	*Markowitz [30]	Review
18	Mason et al. [31]	Different fluoride concentration in the test and comparison toothpastes
19	Parkinson et al. [32]	*In vitro* study
20	Pearce et al. [33]	Different fluoride concentrations and compounds in the test and comparison toothpastes
21	*Pol et al. [34]	Unobtainable article (review?)
22	Ross [35]	No control (placebo/active) group
23	Shapiro et al. [36]	Abstract only
24	Shapiro et al. [37]	The outcome measurements were not clear
25	Stazen and Forman [38]	*In vitro* study
26	Surdacka et al. [39]	*In vitro* study
27	*Tarbet et al. [40]	Study duration was only 4 weeks
28	Uchida et al. [41]	Study on the efficacy of SrCl_2_ in the management of DH following periodontal surgery
29	*West [42]	Different fluoride concentrations and compounds in the test and comparison toothpastes
30	West et al. [43]	Abstract
31	Collins and Perkins [44]	Different compounds in the test and comparison toothpastes. No strontium-free placebo
32	Kumar et al. [45]	Different compounds in the test and comparison toothpastes. No strontium-free placebo

*Studies contain both Strontium and Potassium together.

**Table 2:** Characteristics of potassium based toothpastes excluded studies (reasons).

No.	Study	Reason for exclusion
1	Ajcharanukul et al. [46]	KCl solution was used
2	Andreana et al. [47]	Abstract only
3	Aris et al. [48]	Abstract only
4	Ayad et al. [49]	Different fluoride concentrations and compounds in the test and comparison toothpastes
5	Ayad et al. [50]	Different fluoride concentrations and compounds in the test and comparison toothpastes
6	Bohen and Lafont [51]	In-office treatment
7	Browning et al. [52]	Study on the safety and efficacy of a night guard bleaching agent containing NaF and KNO_3_
8	Charig et al. [53]	Abstract only
9	Conforti et al. [54]	Study duration: 14 days (2 weeks) Different fluoride concentrations and compounds in the test and comparison toothpastes
10	Cooley and Sandoval [55]	Potassium oxalate solution was used
11	Docimo et al. [56]	Different fluoride concentrations and compounds in the test and comparison toothpastes
12	Frechoso et al. [57]	Study duration was 14 days (2 weeks)
13	Gillam et al. [58]	KNO_3_ mouthwash was used
14	Gillam et al. [59]	Different fluoride concentrations and compounds in the test and comparison toothpastes
15	Goncalves et al. [60]	Abstract only
16	Hall et al. [61]	Abstract only
17	Van Haywood et al. [62]	Study on the efficacy of KNO_3_—F gel to reduce bleaching sensitivity
18	Haywood et al. [63]	Study on the efficacy of KNO_3_ toothpaste to reduce bleaching sensitivity
19	Hodosh [64]	Review
20	Hodosh [65]	Not an RCT
21	Jalalian et al. [66]	Study on the efficacy of KNO_3_ in reduction of hypersensitivity in teeth with full-crown preparations
22	Kawamata et al. [67]	Abstract only
23	Lecointre et al. [68]	Different fluoride concentrations and compounds in the test and comparison toothpastes. Study duration: 4 weeks
24	Manochehr-pour et al. [69]	Incomplete data
25	McCormack and Davies. [70]	Review
26	Mordan et al. [71]	Abstract only
27	Morris et al. [72]	Abstract only
28	Orchardson and Gillam [3]	Review
29	Pamir et al. [73]	In-office treatment
30	Peacock and Orchardson [74]	Animal (rat) study for the effect of (K) ions on action potential conduction in A- and C-fibres
31	Peacock and Orchardson [75]	Animal (rat) study to assess the ability of some organic (K) salts to block action potential conduction
32	Pereira et al. [76]	*In vitro* study
33	Pillon et al. [77]	Study on the effect of a single application of 3% potassium oxalate gel immediately after subgingival scaling and root planning on DH
34	Pol et al. [78]	Review
35	Poulsen et al. [9]	Review
36	Prasad et al. [79]	Different fluoride concentrations and compounds in the test and comparison toothpastes
37	Reinhart et al. [80]	A pilot study
38	Salian et al. [81]	Study duration: 4 weeks
39	Salvato et al. [82]	Different fluoride concentrations and compounds in the test and comparison toothpastes
40	Sharma [83]*	No data available at 6 to 8 weeks
41	Sharma et al. [84]	No data available at 6 to 8 weeks
42	Silverman [85]	Test and control toothpastes were not clearly detailed
43	Silverman et al. [86]	Incomplete data
44	Sowinski et al. [87]	No potassium-free comparison group; different fluoride concentrations and compounds in the test and comparison groups
45	Sowinski et al. [88]	Different fluoride concentrations and compounds in the test and comparison toothpastes
46	Stead et al. [89]	Review (mathematical model)
47	Tarbet et al. [90]	Not clear if the study was randomized. Study duration: 4 weeks
48	Tarbet et al. [91]	Study on the pulpal effects of brushing with a (5% KNO_3_) paste used for desensitization
49	Touyz and Stern [92]	KNO_3_ solution was used to reduce DH after periodontal surgery
50	Wang et al. [93]	Abstract only
51	Wara-aswapati et al. [94]	No data available at 6 to 8 weeks
52	Yates et al. [95]	Potassium citrate-containing mouth rinse was used
53	Yates et al. [96]	No potassium-free comparison group
54	Orsini et al. [97]	Different fluoride concentrations and compounds in the test and comparison toothpastes
55	Pradeep and Sharma [98]	Different compounds in the test and comparison toothpastes. Calcium sodium phosphosilicate was evaluated against a potassium nitrate and placebo with no calcium sodium phosphosilicate

*http://www.oralscience.ca/. A randomized parallel group clinical study accessed 2010.

**Table 3:** Characteristics of the included Strontium containing toothpaste studies.

No.	Study	Methods	Participants	Interventions	Outcomes	Results
1	Minkoff and Axelrod [99]*	12 weeks, parallel, double-blind, randomised	57 completing out of 61	10% SrCl_2_ versus 0% SrCl_2_	Tactile and thermal	SrCl_2_ > placebo control (*P* < 0.05) after 4 weeks (subjective), after 8 weeks (air blast), and at 12 weeks (tactile)

*Formulation was subsequently changed from an SrCl_2 _with diatomaceous earth to SrCl_2 _with a silica abrasive/filler. A Strontium chloride toothpaste is no longer available in some markets. A Strontium acetate with fluoride toothpaste is currently available.

**Table 4:** Characteristics of Potassium containing toothpastes included studies.

No.	Study	Methods	Participants	Interventions	Outcomes	Results
1	Nagata et al. [100]	12 weeks, parallel, double-blind, randomised	36 completing out of 36	5% KNO_3 _versus 0% KNO_3_	Tactile, air blast, and subjective	5% KNO3 > control (*P*, 0.05) at 4, 8, and 12 weeks
2	Schiff et al. [101]	12 weeks, parallel, double-blind, randomised	58 completing out of 67	5% KNO_3_ and 0.243% sodium MFP versus 0% KNO_3_ and 0.243% sodium MFP	Thermal, tactile, air blast, and subjective	Test > control (*P* < 0.01) at 6 and 12 weeks
3	Schiff et al. [102]	8 weeks, parallel, double-blind, randomised	39 completing out of 48	5% KNO_3_ and 1500 ppm MFP versus 0% KNO_3_ and 1500 ppm MFP	Tactile and air blast	5% KNO3 > control (*P* < 0.0001) at 4 and 8 weeks
4	Schiff et al. [103]	8 weeks, parallel, double-blind, randomised	80 participants	5% KNO_3_ and 0.243% NaF versus 0.243% NaF	Tactile and air blast	5% KNO3 > positive and negative controls (*P* < 0.05) in tactile and air blast sensitivity, at 4 and 8 weeks
5	Silverman et al. [104]	8 weeks, parallel, double-blind, randomised	110 completing	5% KNO_3_ versus 0% KNO_3_	Tactile, cold air, and subjective	5% KNO3 > +/− F > F control at 4 and 8 weeks (*P* < 0.02); NS between 10% Sr Cl2 and control; 5% KNO3 > +/− F > 10% Sr Cl2 at 8 weeks (*P* < 0.05)
6	Sowinski et al. [105]*	8 weeks, parallel, double-blind, randomised	67 completing	5% KNO_3_ and 0.243% NaF versus 0.243% NaF	Tactile and air blast	KNO_3_ > control significant improvements in tactile and air blast at 4 and 8 weeks

*Product withdrawn from the market.

**Table 5:** Study duration of included studies.

Study	Study duration
Minkoff and Axelrod [99]	12 weeks
Nagata et al. [100]	12 weeks
Schiff et al. [101]	12 weeks
Pradeep and Sharma [98]	8 weeks
Schiff et al. [102]	8 weeks
Schiff et al. [103]	8 weeks
Sowinski et al. [105]	8 weeks

**Table 6:** Statistical tests used in the included studies.

Study	Statistical test
Minkoff and Axelrod [99]	ANOVA, *t*-test, Spearman's rank correlation coefficient
Nagata et al. [100]	Mann-Whitney *U* test Chi-square/Fisher's exact probability test
Schiff et al. [101–103]	ANOVA *t*-test
Silverman et al. [104]	ANOVA
Sowinski et al. [105]	*t*-test

**Table 7:** Number of dropout participants from the included studies and the reasons for dropout (4 studies).

Study	No. of dropouts	Reason(s) for dropping out
Minkoff and Axelrod [99]	4 out of 61	3 had minor side effects upon use of active product 1 for personal reasons
Schiff et al. [101]	9 out of 67	For reasons unrelated to dentifrice use
Schiff et al. [102]	9 out of 48	For reasons unrelated to dentifrice use
Sowinski et al. [105]	All participants completed	No dropouts recorded

**Table 8:** The different types of DH assessment used in the included studies.

Study	DH assessment method(s)	Subjective assessment
Minkoff and Axelrod [99]	Tactile and thermal	Questionnaire
Nagata et al. [100]	Tactile and air blast	Tarbet's scale and questionnaire [106]
Schiff et al. [101]	Tactile, air blast, and thermodontic stimulator (thermal)	VAS
Schiff et al. [102, 103]	Tactile and air blast	Schiff's sensitivity scale (0–3)
Silverman et al. [104]	Tactile and air blast	VAS
Sowinski et al. [105]	Tactile and air blast	Schiff's sensitivity scale (0–3)
